# Supplementary material for: Patterns of Intron Gain and Loss in Fungi
Source: PLoS Biol. 2004 Nov 30;2(12):e422. doi: 10.1371/journal.pbio.0020422 (PMC532390; doi:10.1371/journal.pbio.0020422)
Supplement: Table S1 — Also available at http://genes.mit.edu/NielsenEtAl/. (4.3 MB ZIP). [file pbio.0020422.st001.zip › NielsenEtAl/html/624.html]

AN4869.1.NCU10061.1.MG01153.1.FG10255.1


```
 CLUSTAL W (1.82) Multiple Sequence Alignments - Introns Inserted


Sequence 1: NCU10061.1	254 aa
Sequence 2: FG10255.1	253 aa
Sequence 3: MG01153.1	254 aa
Sequence 4: AN4869.1	259 aa
Alignment Length: 259 aa
Number Identitical Residues: 193 aa
Alignment Score (without introns) 8108


MG01153.1 	MSG1S~AGYDRHITIFSDRGRLYQV-~----E1YAFKAITAANIMAVGVRGKDCAVVLSQ
NCU10061.1	MSG1S~AGYDRHITIFSEQGRLYQV-~----E1YAFKAITAANIMSLGVRGKDCAVVLSQ
FG10255.1 	MS-~A1GAYDRHITIFADNGRLYQV-~----E1YAFKAITAANIMSVGVRGKDCAVVLSQ
AN4869.1  	MSG1S~AGYDRHITIFSDQGRLYQVG1VPKLE~YAFKAITAANITSIGVRGKDCAVVLSQ
          	**. : ..********::.******.  .. * *********** ::*************

MG01153.1 	KKVP0DKLIDPSSVSHIFQISPKVGCVITGSIADARAFAQRAQGEAAEFRYKYGYEMPCD
NCU10061.1	KKVP0DKLIDPSSVSHIFQISPSVGCVITGSIADARAFAQRAQGEAAEFRYKFGYEMPCD
FG10255.1 	KKVP0DKLMDPASVTHIFQLSPSVGCVITGSIADARAFAQRAQGEAAEFRYKYGYEMPAD
AN4869.1  	KKVA0DKLIDPSSVTHIFRLSPSVGCVMTGSIADARASVDRARGEAAEFRYKYGYEMPCD
          	***. ***:**:**:***::**.****:********* .:**:*********:*****.*

MG01153.1 	ALAKRLANISQVYTQR0AYMRPYGIATTLVSVDEEYGPQLFKCDPAGYYTGYKGTAAGPK
NCU10061.1	ALAKRIANISQVYTQR0AFMRPYGVAVTLISVDSEFGPQLFKCDPAGYYTGYKGTAAGPK
FG10255.1 	ALAKRLANISQVYTQR0AYMRPYGVATTLISLDSEYGPQLFKCDPAGYYIGYKGTAAGPK
AN4869.1  	VLAKRLANINQVYTQR0AYMRPLGVAITLCSVDSEKGPQLYKCDPAGYYAGYKATASGPK
          	.****:***.****** *:*** *:* ** *:*.* ****:******** ***.**:***

MG01153.1 	QQEALNHLEKKLKNKDYAEGNWEEVVELAITTLSTVLSMDFKKTEIEIGIVGGPRLDGQE
NCU10061.1	QQEALNHLEKKLKNKDHAPGDWKEVVELAITTLSTVLSMDFKKTEIEIGIVGGPRPDGKE
FG10255.1 	QQEALNHLEKKLRNKEHAEGSWEEVVELAITTLSTVLSMDFKKGEIEIGIVGGPRPEGQE
AN4869.1  	QQEALNHLEKKLKNKDYAEGSWEEVVELGITALSSVLSVDFKKHELEIGIAGGPRTDGKE
          	************:**::* *.*:*****.**:**:***:**** *:****.**** :*:*

MG01153.1 	GTNPNFRKLTEDEIDERLQAIAEKD
NCU10061.1	GTYPGFRTLTEDEIDERLQAIAEKD
FG10255.1 	GTYSGFRTLTEDEIDDRLQAIAEKD
AN4869.1  	GTDPHFRALTEEEIDERLQAIAEKD
          	** . ** ***:***:*********
```
